# Histone H3K9 Trimethylase Eggless Controls Germline Stem Cell Maintenance and Differentiation

**DOI:** 10.1371/journal.pgen.1002426

**Published:** 2011-12-22

**Authors:** Xiaoxi Wang, Lei Pan, Su Wang, Jian Zhou, William McDowell, Jungeun Park, Jeff Haug, Karen Staehling, Hong Tang, Ting Xie

**Affiliations:** 1 Stowers Institute for Medical Research, Kansas City, Missouri, United States of America; 2 The Key Laboratory of Infection and Immunity, Institute of Biophysics, Chinese Academy of Sciences, Beijing, China; 3 Department of Anatomy and Cell Biology, University of Kansas School of Medicine, Kansas City, Kansas, United States of America; Harvard Medical School, Howard Hughes Medical Institute, United States of America

## Abstract

Epigenetic regulation plays critical roles in the regulation of cell proliferation, fate determination, and survival. It has been shown to control self-renewal and lineage differentiation of embryonic stem cells. However, epigenetic regulation of adult stem cell function remains poorly defined. *Drosophila* ovarian germline stem cells (GSCs) are a productive adult stem cell system for revealing regulatory mechanisms controlling self-renewal and differentiation. In this study, we show that Eggless (Egg), a H3K9 methyltransferase in *Drosophila*, is required in GSCs for controlling self-renewal and in escort cells for regulating germ cell differentiation. *egg* mutant ovaries primarily exhibit germ cell differentiation defects in young females and gradually lose GSCs with time, indicating that Egg regulates both germ cell maintenance and differentiation. Marked mutant *egg* GSCs lack expression of trimethylated H3K9 (H3k9me3) and are rapidly lost from the niche, but their mutant progeny can still differentiate into 16-cell cysts, indicating that Egg is required intrinsically to control GSC self-renewal but not differentiation. Interestingly, BMP-mediated transcriptional repression of differentiation factor *bam* in marked *egg* mutant GSCs remains normal, indicating that Egg is dispensable for BMP signaling in GSCs. Normally, Bam and Bgcn interact with each other to promote GSC differentiation. Interestingly, marked double mutant *egg bgcn* GSCs are still lost, but their progeny are able to differentiate into 16-cell cysts though *bgcn* mutant GSCs normally do not differentiate, indicating that Egg intrinsically controls GSC self-renewal through repressing a Bam/Bgcn-independent pathway. Surprisingly, RNAi-mediated *egg* knockdown in escort cells leads to their gradual loss and a germ cell differentiation defect. The germ cell differentiation defect is at least in part attributed to an increase in BMP signaling in the germ cell differentiation niche. Therefore, this study has revealed the essential roles of histone H3K9 trimethylation in controlling stem cell maintenance and differentiation through distinct mechanisms.

## Author Summary

Epigenetic regulation plays critical roles in the regulation of cell proliferation, fate determination, and survival. It has been extensively studied in embryonic stem cells for its roles in the control of self-renewal and lineage differentiation. However, epigenetic regulation of adult stem cell function remains poorly defined. In this study, we show that Eggless (Egg), a H3K9 methyltransferase in *Drosophila*, is required in germline stem cells (GSCs) for controlling self-renewal and in escort cells for regulating germ cell differentiation. *egg* mutant ovaries exhibit both germ cell differentiation defects and GSC loss, indicating that Egg regulates both germ cell maintenance and differentiation. Intrinsic inactivation of *egg* function in GSCs leads to loss of trimethylated H3K9 expression and rapid departure from the niche, indicating that Egg is required intrinsically to control GSC self-renewal. Our genetic results reveal that Egg intrinsically controls GSC self-renewal through repressing a Bam/Bgcn-independent pathway. Furthermore, RNAi-mediated *egg* knockdown in escort cells leads to germ cell differentiation defects due to increased BMP signaling. Therefore, this study has revealed essential roles of histone H3K9 trimethylation in controlling stem cell maintenance and differentiation through distinct mechanisms in the *Drosophila* ovary.

## Introduction

Histone modification represents one of the most common epigenetic mechanisms for controlling gene expression, and thus cell proliferation, fate determination and survival during development [1]. Histone modification has recently been subjected to extensive investigation for its roles in the control of self-renewal and lineage differentiation of embryonic stem cells (ESCs) by disrupting functions of the enzymes that are important for catalyzing the modifications [2]–[7]. Among different histone modifications, trimethylation of histone 3 lysine 9 (H3K9me3) has been widely studied and is often associated with heterochromatin formation, gene repression and transcriptional elongation in different tissue types and organisms [1]. SETDB1, one of the H3K9 trimethylases in the mouse, was recently shown to be important for maintaining ESC self-renewal [8]. However, its role in adult stem cell regulation remains to be determined.

In the *Drosophila* ovary, two or three GSCs are located at the tip of the germarium, which is the structure located at the apical end of an ovariole [9], [10]. These GSCs physically interact with cap cells anteriorly and escort cells laterally. The immediate differentiating GSC progeny, known as cystoblasts (CBs), can further divide synchronously without cytokinesis to form 2-cell, 4-cell, 8-cell and 16-cell cysts. CBs, mitotic cysts and newly formed 16-cell cysts are surrounded by escort cells. Cap cells form a niche for maintaining GSC self-renewal by producing BMP-like molecules Dpp and Gbb [11]–[13]. Dpp and Gbb activate BMP signaling in the GSC to directly repress expression of differentiation factors such as *bam*, and thereby maintain GSC self-renewal [13], [14]. Chromatin remodeling factors, such as ISWI and Stonewall, have been shown to be important for maintaining GSC self-renewal through distinct mechanisms. ISWI is required for repressing *bam* transcription in GSCs [15], while Stonewall likely represses a Bam-independent pathway [16]. Lsd1 is a H3K4 demethylase in the *Drosophila* ovary, and its mutations cause upregulation of H3K4 trimethylation and gene activation [17]. Recently, Lsd1 has been shown to be required in escort cells (ECs) to repress *dpp* expression and promote germ cell differentiation [18]. These findings indicate that epigenetic regulation is important for GSC self-renewal.

In *Drosophila*, there are three known H3K9 methyltransferases, *Su(var)3-9*, *G9a* and *eggless* (*egg*, also known as *dSETDB1*). *Su(var)3-9* was the first identified H3K9 methyltransferase in *Drosophila* [19], and it is responsible for H3K9me3 at the core of the chromocenter, which provides docking sites for HP1 recruitment and thus heterochromatin formation and maintenance [20], [21]. Recently, *G9a* was also shown to exhibit H3K9-, H3K27- and H3K4- methyltransferase activity and localize to the euchromatic region, but it is dispensable for normal *Drosophila* development [22], [23]. Egg is the histone methyltransferase responsible for maintaining H3K9me3 on the fourth chromosome, and it works with Su(var)3-9 to maintain H3K9me3 in the pericentric heterochromatin of all chromosomes [24]–[27]. Egg is expressed throughout *Drosophila* development, and is an essential gene because its homozygous deletion causes lethality [25], [26]. In addition, the females carrying homozygous EMS-induced *egg* mutations do not lay any eggs, and it is this phenotype upon which its name is based [28]. The *egg* mutant ovaries exhibit defects in follicle cell proliferation and the maintenance or survival of somatic cells and germ cells [28]. Consistently, Windei (Wde), the *Drosophila* homolog of human MCAF1, is an essential Egg cofactor and is also required for germ cell maintenance [29]. Interestingly, Egg is located in pericentric heterochromatin and catalyzes H3K9 trimethylation in GSCs and their immediate descendants, while SU(VAR)3-9 is primarily in charge of H3K9 trimethylation in more differentiated germline cysts in egg chambers [27]. Mutations in *egg* and *Su(var)3-9* abolish H3K9me3 from germ cells in the germarium and the developing egg chambers, respectively. Although *egg* is proposed to maintain the survival of germ cells in the *Drosophila* ovary, it remains unclear whether it is required for GSC maintenance or simply germ cell survival. In this study, we have revealed the essential role of Egg in controlling GSC self-renewal and a novel role of Egg in the regulation of germ cell differentiation.

## Results

### Egg Is Essential for GSC Maintenance and Germ Cell Differentiation

**Figure 1:**
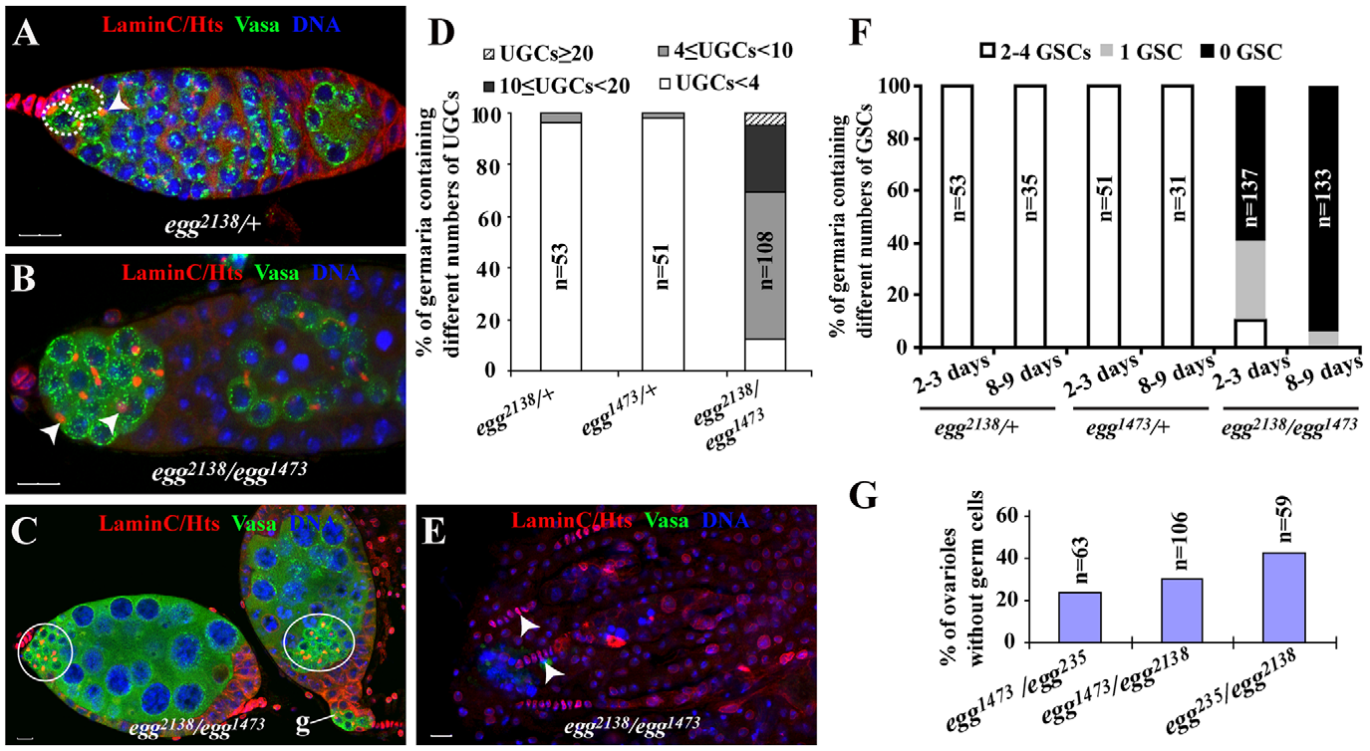
*egg* is required for GSC maintenance and germ cell differentiation. Control and *egg* mutant germaria are labeled for Lamin C (red, terminal filament and cap cells), Hts (red, spectrosomes and fusomes), Vasa (green, germline cells) and DAPI (blue, nuclei). (A) A week-old *egg* heterozygous control germarium showing two GSCs (broken circles) and one CB (arrowhead). (B) 3-day-old mutant *egg* germarium shows the accumulation of spectrosome-containing undifferentiated germ cells (UGCs) (arrowheads), indicative of differentiation defects. (C) The *egg* mutant chambers following the germarium (indicated by g) contain both differentiated germ cells and a cluster of undifferentiated spectrosome-containing single germ cells (circled). (D) 3-day-old *egg* mutant germaria contain more UGCs than the heterozygous controls. Germaria are categorized based on the UGC number. “n” indicates the number of germaria examined for each genotype. (E) Week-old *egg* mutant germaria showing only remaining TFs (arrowheads) and complete absence of germ cells including GSCs, which is indicated by absence of Vasa expression. (F) GSCs are gradually lost in *egg* mutant germaria. Germaria are categorized by the number of GSCs per germarium. (G) Germless ovarioles in 3–5 day-old ovaries of different *egg* mutant combinations are consistently observed. Scale bars represent 10 µm.

To investigate how germ cells are lost in *egg* mutant ovaries, we examined the germ cell phenotypes of different *egg* mutant allelic combinations. In the *Drosophila* ovary, somatic cells and different germ cell types can be distinguished using molecular markers. Lamin C can label both TFs and cap cells, which can be easily distinguished by their distinct cellular morphologies [12]: TF cells are disc-like cells lining up in a row, while cap cells are round-shaped cells tightly packing together next to TFs. Vasa can label all the germ cells including GSCs [30], [31], while Hts labels spectrosomes in GSCs and CBs as well as branched fusomes in cystocytes [32] (Figure 1A). In this study, three *egg* mutants, *egg^2138^* and *egg^1473^* and *egg^235^*, were used to investigate its function in female germ cell development. The Egg protein contains two tudor domains and one bifurcated SET domain, which carry out the functions of binding to the methylated H3K9 and catalyzing H3K9 trimethylation, respectively [28]. The mutations in *egg^1473^* and *egg^235^* were previously shown to cause the production of truncated proteins by deletion of the entire SET domain and all the functional domains, respectively (Clough et al., 2007). The *egg^2138^* mutation corresponds to a truncated protein resulting from deletion of the SET domain and part of the second tudor domain and thus is a strong mutation (T. Hazelrigg, personal communications). Heterozygous *egg^2138^* and *egg^1473^* mutant germaria carry two or three GSCs and one CB as in normal wild-type germaria (Figure 1A and 1F). In contrast, *egg^2138^*/*egg^1473^*, *egg^2138^*/*egg^235^*, *egg^1473^*/*egg^235^* mutant germaria exhibit two defects in germ cell development in addition to the previously reported follicle cell defects. The germaria in the newly eclosed two to three-day-old mutant females contain many spectrosome-containing single germ cells, which are located away from cap cells (Figure 1B). The undifferentiated GSC-like or CB-like cells also persist after they have been packed into egg chambers along with differentiated germ cell cysts (Figure 1C). For quantification, undifferentiated GSC-like or CB-like cells located outside the niche in the germarium are quantified as undifferentiated germ cells (UGCs). For the heterozygous controls, over 95% of the germaria contain one, two or three spectrosome-containing UGCs (Figure 1D). By contrast, 88% of the *egg^2138^*/*egg^1473^* mutant germaria harbor more than 4 UGCs, and 30% of them have ten or more UGCs (Figure 1D). These results have revealed that *egg* is required for germ cell differentiation.

As reported previously, *egg* is also required for germ cell maintenance [29]. Indeed, *egg* mutant germaria gradually lose their GSCs and eventually become agametic with time (Figure 1E and 1F). Even at the age of 2 or 3 days, 59% of the *egg^2138^*/*egg^1473^* mutant germaria contain no GSCs, and 16% of them completely lose germ cells, including GSCs, indicating that some GSCs have already been lost in young mutant females (Figure 1F). At the age of 8 or 9 days, the GSC loss phenotype becomes more severe. 94% of those mutant germaria contain no GSCs, and 84% of them become completely depleted of germ cells (Figure 1F). Regarding the GSC loss phenotype, other mutant allelic combinations give the comparable GSC loss phenotype at the age of one week old (Figure 1G). These results show that *egg* is required for GSC maintenance.

### Egg Is Required Intrinsically for GSC Self-Renewal and Proliferation

**Figure 2:**
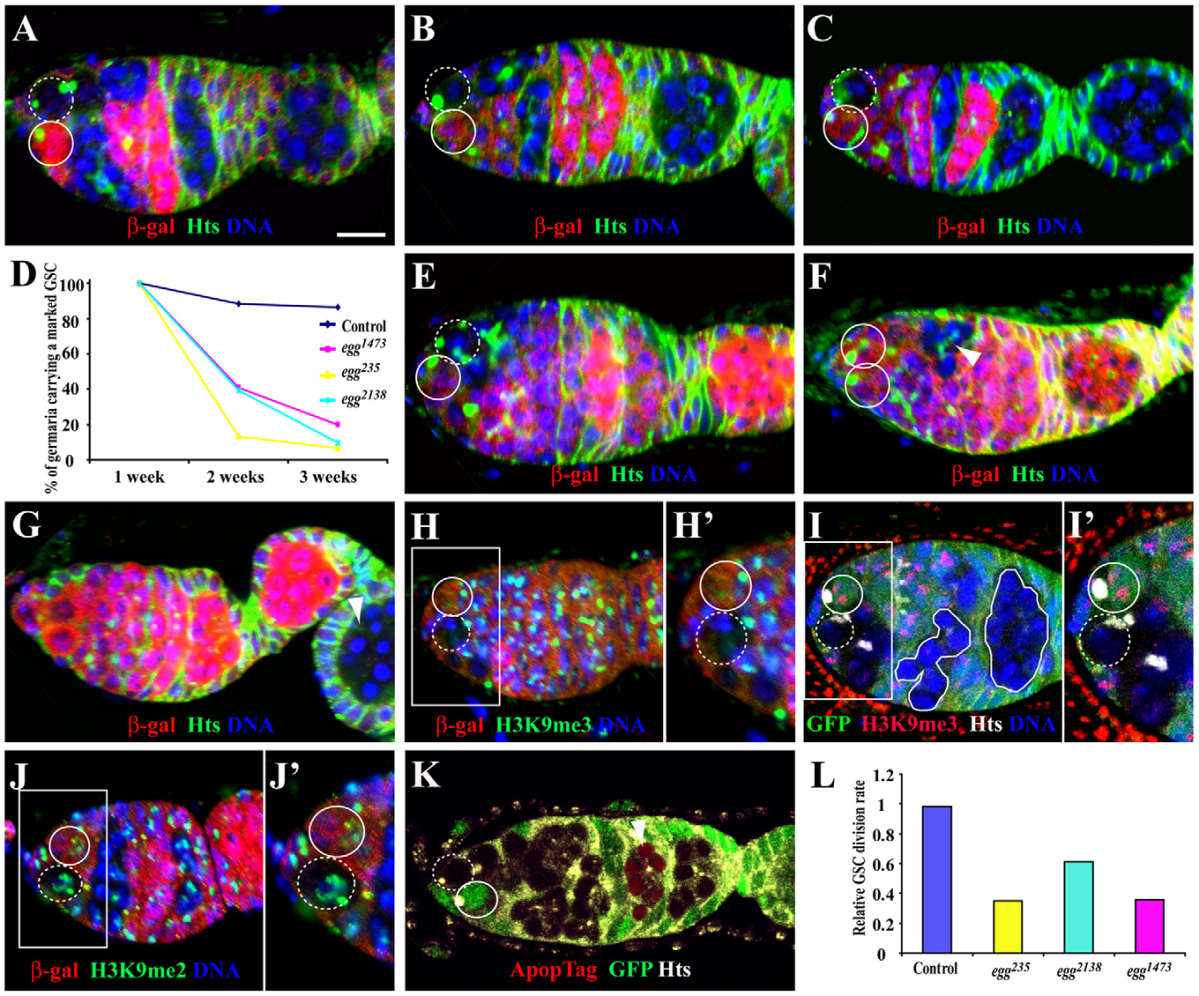
*egg* is required intrinsically for GSC maintenance and the survival of late differentiated germ cells. Germaria in A–G are labeled for LacZ (red), Hts (green) and DAPI (blue), while unmarked and marked GSCs are indicated by broken and solid circles, respectively. (A–C) Marked wild-type GSCs are maintained one week (A), two weeks (B) and three weeks (C) after clonal induction (ACI). (D) Percentages of the germaria carrying a marked wild-type or *egg* mutant GSC clone change with time (one week, two weeks and three weeks ACI). (E) A marked *egg* mutant GSC still remains in the niche one week ACI. (F, G) The marked mutant GSC is lost from the niche two weeks (F) or three weeks (G) ACI, , which is evidenced by presence of a marked mutant cyst (arrowheads in F and G). (H, H′) A marked *egg* mutant GSC (broken circle) has lost H3K9me3 staining in comparison with its neighboring unmarked control GSC (circle) one week ACI. (I, I′) A marked *egg* mutant GSC (broken circle) and mutant 16-cell cysts (solid lines) have no H3K9me3 staining in comparison with its neighboring unmarked control GSC (circle) and cysts twelve days ACI. (J, J′) Marked *egg* mutant GSC (broken circle) and unmarked control GSC (solid circle) have comparable H3K9me2 staining one week ACI. (K) A marked *egg* mutant 16-cell cyst (arrowhead), but not the marked *egg* mutant GSC (broken circle), is apoptotic twelve days ACI. (L) Relative GSC division rates for marked control and *egg* mutant GSCs. Scale bar: 10 µm.

The genes identified so far for GSC regulation are required for either GSC maintenance or differentiation but rarely for both. To further understand how Egg regulates both GSC maintenance and differentiation, we used FLP-mediated FRT recombination to remove *egg* function intrinsically from *arm-lacZ*-marked GSCs. In this study, marked GSCs are identified by loss of *lacZ* (encoding β-galactosidase or β-gal) expression, presence of spectrosome and physical contact with cap cells/the niche as in our previous studies [11], [33]. As shown previously, about 80% of the marked control GSCs detected at the first week after clonal induction (ACI) are still maintained three weeks ACI (Figure 2A–2D). The marked control GSCs still remain in the niche two weeks and three weeks ACI (Figure 2B and 2C). In contrast, less than 20% of the marked *egg* mutant GSCs for *egg^1473^*, *egg^235^* and *egg^2138^* detected at the first week ACI are still maintained three weeks ACI, indicating that Egg is required intrinsically for maintaining GSCs (Figure 2D–2G). Two or three weeks ACI, the lost marked mutant egg GSCs have developed into differentiated 16-cell germline cysts either in the germaria or egg chambers (Figure 2F and 2G). The differentiation of marked *egg* mutant cysts could be due to perdurance of Egg protein. To rule out this possibility, we examined H3K9me3 in marked *egg* mutant germline clones. Consistent with the role of Egg in catalyzing H3K9 trimethylation, the marked *egg^1473^*, *egg^235^* or *egg^2138^* mutant GSCs and cysts as early as one week ACI have abolished H3K9 trimethylation in comparison with their neighboring control GSCs and cysts (Figure 2H and 2I), but their H3K9 dimethylation remains unchanged (Figure 2J). These results confirm that Egg is a key enzyme responsible for H3K9 trimethylation in GSCs and early germ cell cysts, and that there is not much Egg protein perdurance in the marked egg mutant germline clones one week ACI. However, almost all the germaria carrying a marked mutant GSC do not accumulate marked *egg* mutant spectrosome-containing single germ cells, indicating that Egg is intrinsically dispensable for germ cell differentiation. These results indicate that Egg is required intrinsically only for controlling GSC maintenance but not for differentiation.

The loss of the marked *egg* mutant GSCs could be due to their competitive disadvantage over their neighboring control GSCs. We used an RNAi-mediated knockdown approach to inactivate *egg* function in all the GSCs in the niche using *nanos-gal4*-driven UAS-RNAi expression. Two independent RNAi lines [*eggRNAi-1* (HMS00443) and *-2* (HMS00112)], which can be expressed in female germ cells including GSCs using *nanos-gal4VP16* [34], were used to knockdown *egg* function in GSCs. Consistent with *egg* mutant clonal analysis results, germline expression of *eggRNAi-1* leads to almost complete elimination of germ cells including GSCs in one-week old females (Figure S1A–S1C). However, germline expression of *eggRNAi-2* results in formation of swollen germaria due to the accumulation of a few more spectrosome-containing single germ cells and differentiated cysts, but only rare GSC loss (Figure S1C–S1E), suggesting that *eggRNAi-2* may be less effective in knocking down *egg* expression. Interestingly, the accumulated single germ cells and germ cell cysts in the germarium are positive for the commonly used DNA damage marker H2AX in comparison with the control that only meiotic germ cells are positive for this marker, indicating that *egg* is involved in DNA damage control. The accumulated DNA damage could also explain the accumulation of some spectrosome-containing single germ cells due to mitotic arrest. These results support the idea that Egg is required intrinsically for maintaining GSCs, and also suggest that it controls GSC maintenance possibly via maintaining the genome integrity.

Loss of the marked mutant *egg* GSCs could be due to either defective self-renewal or apoptosis. To rule out the possibility that the mutant *egg* GSCs are lost due to apoptosis, we performed TUNEL-based ApopTag labeling of the marked mutant *egg* GSCs and cysts. Interestingly, no marked mutant *egg* GSCs are apoptotic (total 38 marked *egg* mutant GSC clones examined), suggesting that DNA damage caused by loss of *egg* function leads to defective GSC self-renewal but not apoptosis (Figure 2K). Interestingly, *egg* mutant mitotic cysts and 16-cell cysts in the regions 1 and 2a are not apoptotic. However, among the 38 ovarioles, 9 of them carry at least one apoptotic *egg* mutant 16-cell cyst in the regions 2b or 3 of the germaria, indicating that DNA damage caused by loss of *egg* function leads to apoptosis of differentiated 16-cell cysts (Figure 2G and 2K). Because DNA damage often results in cell cycle arrest, we would expect that loss of *egg* function slows down proliferation of GSCs, CBs and mitotic cysts. To test this idea, we then determined the relative division rates for marked control and *egg* mutant GSCs as we previously reported [11]. As expected, the relative division rate for marked wild-type control GSCs is close to 1 (Figure 2L). In contrast, the relative division rates for marked *egg* mutant GSCs are much lower than that for the control, indicating that *egg* mutant GSCs divide much slower than wild-type controls (Figure 2L). Consistently, only 24.3% of the marked mutant *egg^1473^* GSCs (n = 33) are positive for BrdU in contrast with 32.8% for the unmarked control GSCs (n = 134) in the same population of the germaria. These results could also explain the accumulation of CBs and mitotic cysts in the germarium following the inactivation of *egg* function in the germline (Figure S1E). These results indicate that Egg is required intrinsically for promoting GSC self-renewal and proliferation.

### Egg Controls GSC Self-Renewal by Repressing a Bam-Independent Differentiation Pathway

**Figure 3:**
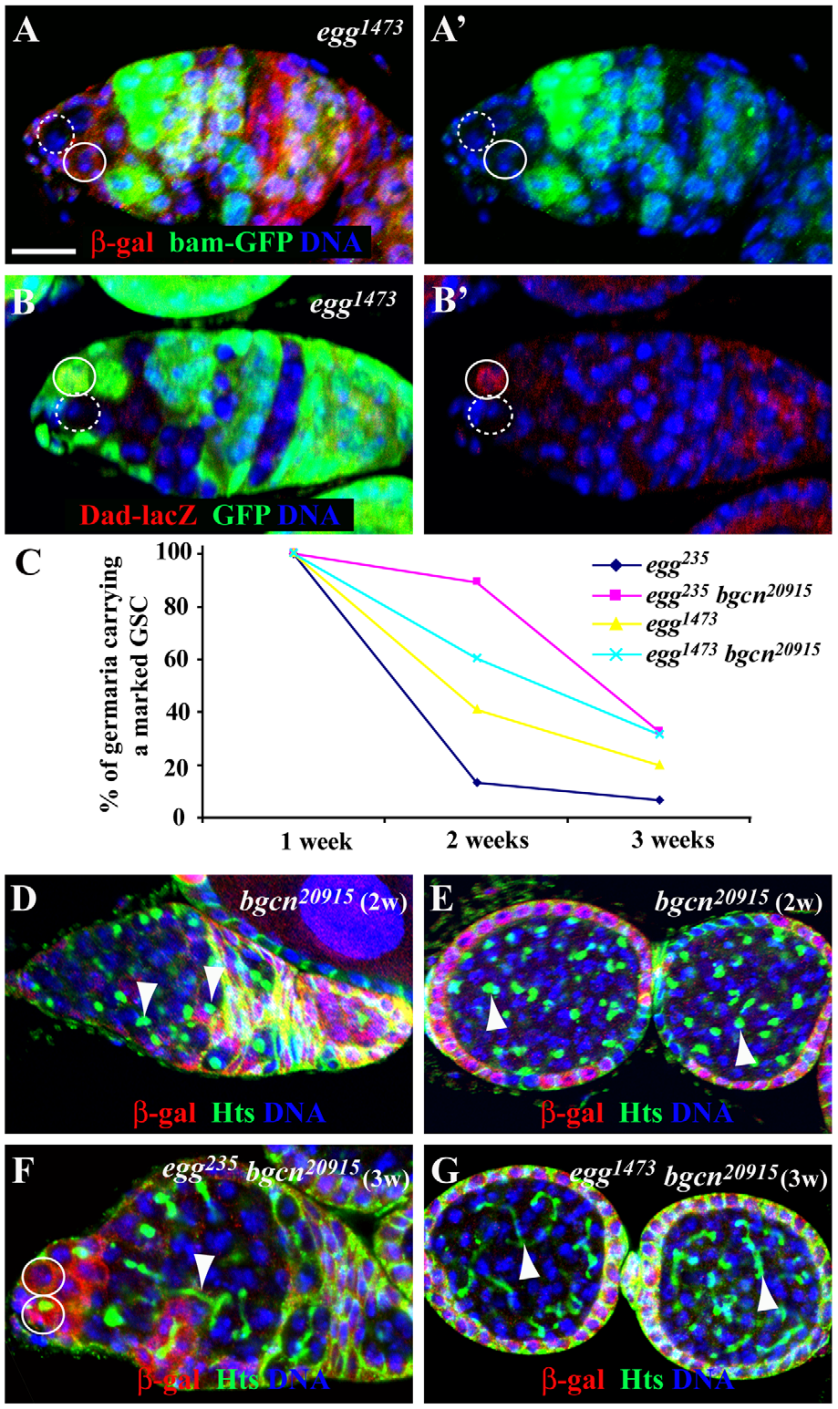
*egg* is required intrinsically for maintaining GSCs by repressing a Bam/Bgcn-independent differentiation pathway. (A and A′) Both *lacZ*-negative marked *egg* mutant GSC (broken circle) and *lacZ*-positive unmarked control GSC repress *bam-GFP* expression (A′). (B and B′) A GFP-negative marked *egg* mutant GSC (broken circle) loses its *Dad-lacZ* expression (B′) in comparison with the neighboring GFP-positive unmarked control GSC (solid circle). (C) Percentages of marked *egg* mutant GSCs and *egg bgcn* double mutant GSCs change with time. (D) The marked *lacZ*-negative *bgcn* mutant GSC continuously generates spectrosome-containing single germ cells (arrowhead). (E) The marked LacZ-negative *bgcn* mutant single germ cells in the two egg chambers contain a spectrosome (arrowhead). (F) The marked *lacZ*-negative *egg bgcn* mutant GSC is lost from the niche evidenced by the presence of two *lacZ*-positive GSCs in the niche, but its marked mutant progeny remaining in the germarium contain a branched fusome (arrowhead). (G) The marked *lacZ*-negative *egg bgcn* mutant germ cells in the two egg chambers have a branched fusome (arrowhead). Scale bar: 10 µm.

Niche-activated BMP signaling is known to be necessary and sufficient for GSC self-renewal [11], [12]. In *Drosophila* ovarian GSCs, active BMP signaling represses *bam* expression and activates *Dad* expression, which can be monitored by reporter lines *bam-GFP* and *Dad-lacZ*, respectively [13], [14], [35], [36]. In the marked mutant *egg^1473^* GSCs, *bam-GFP* is still repressed as in the neighboring unmarked control GSCs of the same germaria, indicating that *egg* is dispensable for BMP signaling-mediated *bam* repression in GSCs (Figure 3A–3A′). Interestingly, in the marked mutant *egg^1473^* GSCs, *Dad-lacZ* fails to be activated to similarly high expression levels as those in their neighboring unmarked control GSCs of the same germaria, indicating that *egg* intrinsically regulates *Dad* transcription in GSCs (Figure 3B–3B′). Because Egg only regulates transcriptional activation of *Dad* but not repression of *bam* in GSCs, we speculate that Egg is dispensable for BMP signaling but indirectly regulates *Dad* expression.

To further investigate if Egg controls GSC self-renewal by repressing a Bam-independent pathway, we generated *lacZ*-marked *egg bgcn* double mutant GSCs and examined their maintenance and differentiation. Mutations in either *bam* or *bgcn* can completely block GSC differentiation, and *bam* overexpression fails to induce GSC differentiation in the absence of *bgcn* function, indicating that *bam* and *bgcn* function in the same genetic pathway to control GSC differentiation [37]–[39]. *Bgcn^20915^* is a strong or null mutation [40]. Interestingly, those *egg bgcn* double mutant GSCs are lost much faster than the marked control GSCs, but slower than the marked *egg* mutant GSCs, indicating that Egg maintains GSC self-renewal at least in part by repressing a Bam/Bgcn-independent pathway (Figure 3C). The partial rescue of the mutant *egg* GSC loss phenotype by the *bgcn* mutation further supports the idea that *egg* is required intrinsically for GSC self-renewal. In contrast with the knowledge that marked *bgcn* mutant GSCs continuously produce spectrosome-containing single germ cells (Figure 3D and 3E), the marked *egg bgcn* mutant GSC progeny can differentiate into cysts in the germarium based on the morphology of their branched fusome three weeks ACI (Figure 3F and 3G). These differentiated double mutant cells with a branched fusome can also be found to be packed together in egg chambers, indicating that these double mutant germ cells do not undergo proper terminal differentiation (Figure 3G). These results suggest that *egg* maintains GSC self-renewal at least partly by repressing a Bam/Bgcn-independent pathway.

### Egg Does Not Intrinsically Regulate E-Cadherin Accumulation in the Stem Cell-Niche Junction

**Figure 4:**
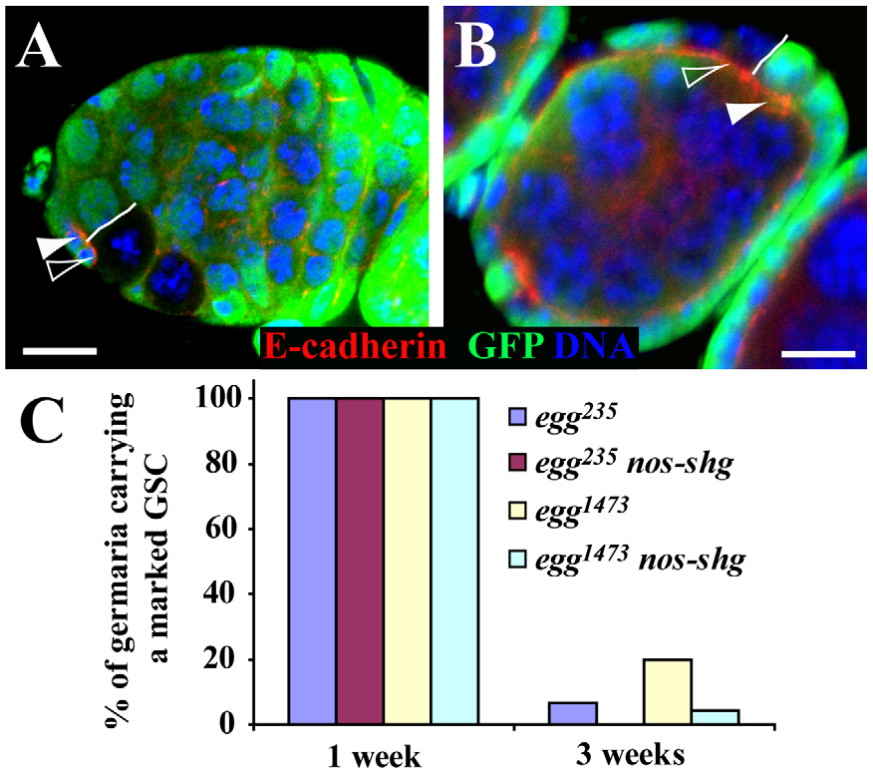
*egg* is dispensable intrinsically for maintaining E-cadherin accumulation in the GSC-niche junction. (A) A GFP-negative marked *egg* mutant GSC (filled arrowhead) has similar E-cadherin accumulation in the GSC-niche junction to its neighboring GFP-positive unmarked control GSC (open arrowhead). The solid line highlights the boundary between the two GSCs. (B) GFP-negative marked *egg* follicle cells (filled arrowhead) have similar apical E-cadherin accumulation to their neighboring GFP-positive unmarked control follicle cells (open arrowhead). The solid line highlights the boundary between the mutant and control follicle cells. (C) *nos-gal4* driven germ cell-specific expression of E-cadherin fails to rescue the stem cell loss phenotype of marked mutant *egg* GSCs. Scale bars: 10 µm.

E-cadherin is required for anchoring GSCs in the niche for long-term self-renewal by accumulating in the stem cell-niche junction [33]. To investigate if Egg is required for regulating E-cadherin accumulation in GSCs, we examined E-cadherin accumulation in the stem cell-niche junction between a marked GSC and its neighboring control GSC. After carefully examining 10 such *egg* mutant and control GSC pairs, we did not observe any difference in E-cadherin accumulation in the stem cell-niche junction between them (Figure 4A). In addition, *egg* mutant and wild-type follicle cells in the egg chamber do not have any obvious difference in E-cadherin accumulation on their apical side (Figure 4B). To further rule out the possibility that E-cadherin is required for Egg-mediated GSC maintenance, we tested if *nanos-gal4*-driven germ cell-specific *UASp-shg* (*shg* encodes E-cadherin in *Drosophila*) expression could rescue or slow down the loss phenotype of the marked *egg* mutant GSCs. *UASp-shg* has been used previously to express E-cadherin in GSCs [40], [41]. Consistent with the idea that Egg does not regulate E-cadherin in GSCs, forced E-cadherin expression shows little rescue effect on the loss phenotype of the mutant *egg* GSCs (Figure 4C). Taken together, we conclude that Egg does not maintain GSCs via regulation of E-cadherin accumulation in the GSC-niche junction.

### Egg Is Required in Escort Cells (ECs) for Controlling the Differentiation of GSC Progeny

**Figure 5:**
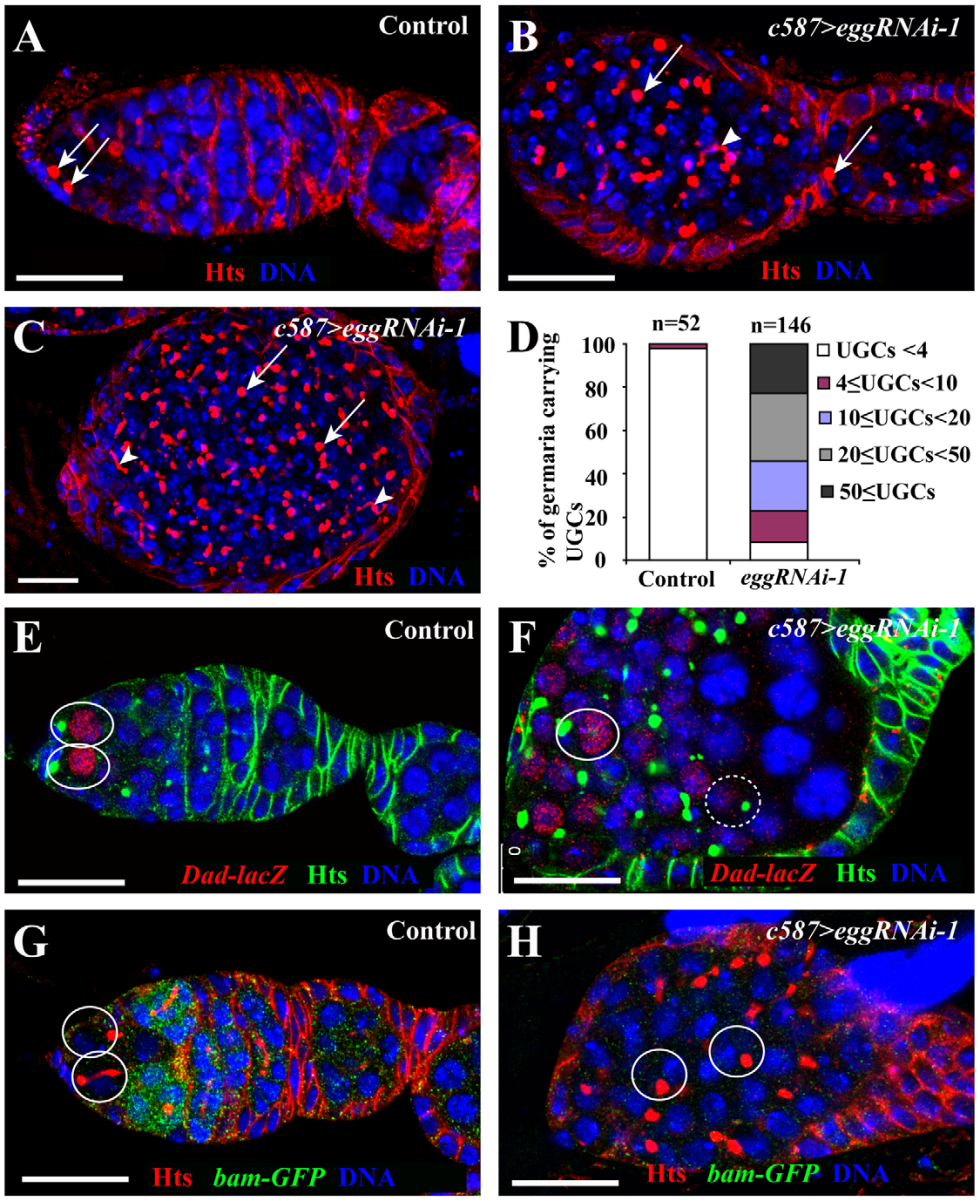
*egg* is required in ECs to control germ cell differentiation. (A) A wild-type germarium showing two GSCs (arrows) tethered to cap cells and one CB containing round spectrosome. (B, C) *C587-gal4* driven *egg* knockdown leads to accumulation of spectrosome-containing cells (arrows) and differentiated cysts containing a branched fusome (arrowhead). (D) Quantification of spectrosome-containing UGCs in week-old *egg* knockdown ovaries. (E) *Dad-lacZ* expression is restricted to GSCs (ovals) in a wild-type germarium. (F) In a *c587-gal4*-driven *egg* knockdown germarium, some spectrosome-containing UGCs (solid circle) express high *Dad-lacZ*, while the others (broken circles) express low *Dad-lacZ*. (G) *bam*-GFP expression is repressed in GSCs (circles) but upregulated in differentiating cysts in a wild-type germarium. (H) In a *c587-gal4*-driven *egg* knockdown germarium, *bam-GFP* expression is absent from spectrosome-containing UGCs (circles). Scale bars: 20 µm.

ECs have recently been shown to control germ cell differentiation by repressing Dally expression through EGFR signaling and thus preventing Dpp diffusion outside the GSC niche [42]–[45]. Thus, we then tested if *egg* function is required in ECs for controlling germ cell differentiation using EC-specific RNAi-mediated knockdown. *C587-gal4* is specifically expressed in ECs and early somatic follicle progenitor cells [13]. To avoid the potential off-target effect of RNAi-mediated knockdown, three more independent RNAi constructs targeting different regions of the *egg* transcript, [*eggRNAi-3*(VDRC#101677), *-4*(VDRC#33730) and *-5*(VDRC#22172)], were used in this study in addition to *eggRNAi-1* and *-2*. In contrast with the ovaries carrying *c587-gal4* or *UAS-RNAi* alone, which contain a germarium followed by a string of egg chambers (Figure 5A), the ovaries carrying both *c587-gal4* and one of the five *UAS-RNAi* constructs for *egg* often have their germaria containing a mixture of spectrosome-containing single germ cells and differentiated germ cell cysts, indicative of germ cell differentiation defects (Figure 5B and 5C; Figure S2). Although the germaria and egg chambers contain differentiated cysts evidenced by the presence of a branched fusome, most of the germ cells are spectrosome-containing single germ cells (Figure 5B–5D). The single germ cells in the germaria fail to differentiate further even after they are packed into individual egg chambers (Figure 5B and 5C; Figure S2). The budding defects observed in *egg* knockdown ovaries are likely caused by disruption of follicle progenitor cell proliferation and differentiation (Figure 5B and 5C; Figure S2). These results demonstrate that *egg* is required in ECs for controlling CB differentiation and in follicle progenitor cells for their proper differentiation or proliferation.

To further determine if single germ cells accumulated in the germaria are GSC-like or CB-like, we examined the expression of *bam-GFP* and *Dad-lacZ*. As mentioned earlier, *Dad-lacZ* is expressed primarily in the GSCs of the control germaria carrying only *c587-gal4* or *UAS-RNAi* (Figure 5E), and *bam-GFP* is normally expressed in differentiated germ cells but is repressed in GSCs (Figure 5G). In contrast, in the germaria in which *egg* function is knocked down in ECs, most of spectrosome-containing single germ cells further away from cap cells still retain *Dad-lacZ* expression and repress *bam-GFP* expression similar to endogenous GSCs, (Figure 5F and 5H). These results indicate that the accumulated single germ cells in the germaria behave like GSCs, and also further suggest that in the absence of *egg* function in ECs, CBs fail to differentiate likely due to upregulation of BMP signaling in germ cells.

### Egg Controls BMP Signaling Activity in Differentiated Germ Cells via ECs

**Figure 6:**
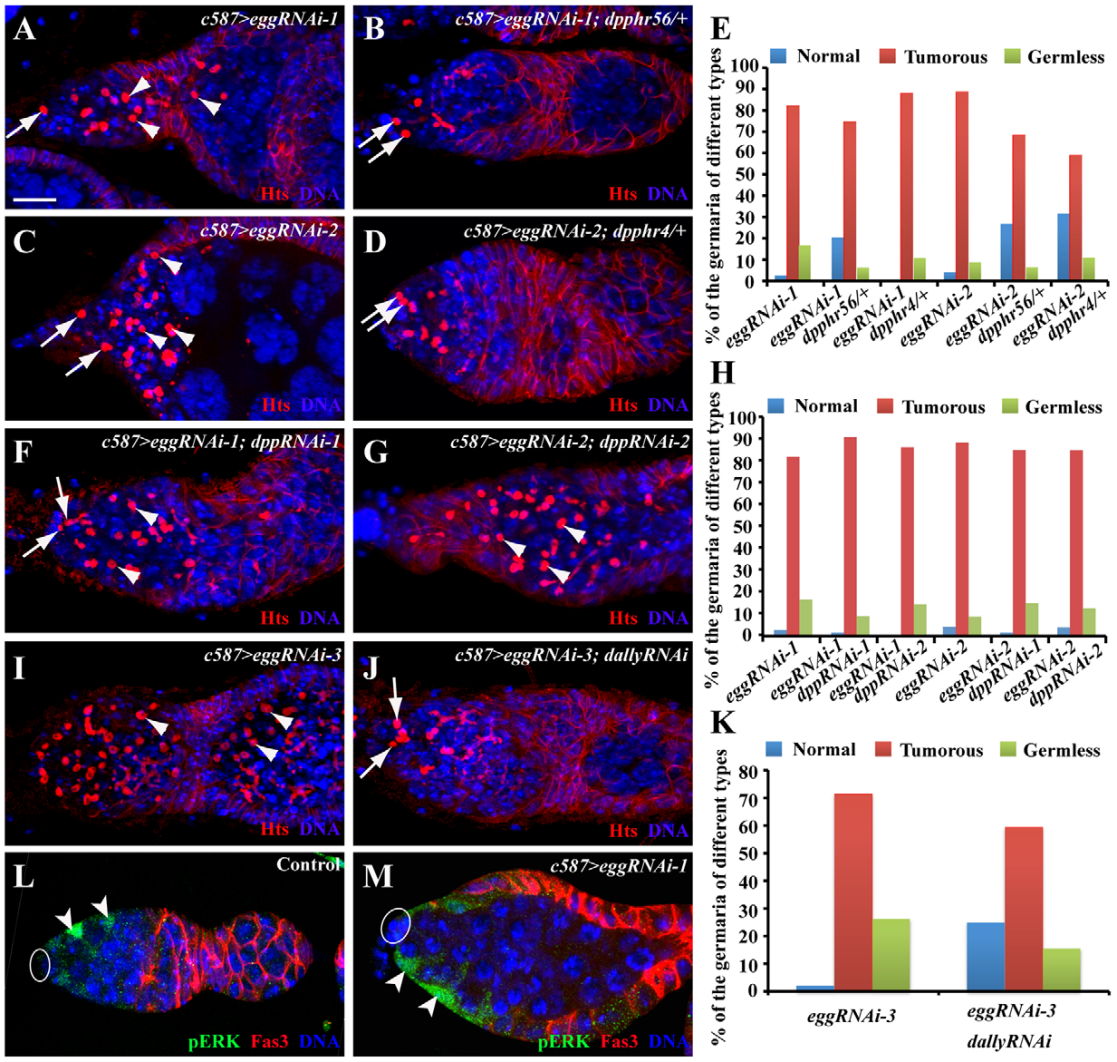
*egg* knockdown in ECs causes EC gradual loss and increases BMP signaling in differentiated germ cells. (A–E) RNAi-mediated knockdown of *egg* causes accumulation of single germ cells containing a spectrosome (arrowhead, A and C) in addition to endogenous GSCs (arrows, A and C), while removal of a copy *dpp* gene using *dpp^hr4^* or *dpp^hr56^* mutation can suppress the germ differentiation defect and thus increase normal germaria with two or three GSCs (arrows) and differentiated germ cells (B and D). The quantitative results are summarized in E. (F–H) RNAi-mediated *dpp* knockdown in ECs fails to suppress the germ cell differentiation defect caused by *egg* knockdown in ECs. Double knockdown germaria still accumulate excess spectrosome-containing single germ cells (arrowheads, F and G) in addition to endogenous GSCs (arrows, G). The quantitative results are summarized in H. (I–K) *dally* knockdown in ECs suppresses the germ cell differentiation defect evidenced by a decrease in spectrosome-containing single germ cells (arrowheads in I; endogenous GSCs indicated by arrows in J). K shows quantitative results. (L, M) *egg* knockdown in ECs (arrowheads, M) does not affect pERK expression in in comparison with control ECs (arrowheads, L). Ovals in L and M indicate cap cells. Scale bars: 10 µm.

To investigate if increased BMP signaling activity is responsible for the germ cell differentiation defect caused by *egg* knockdown in ECs, we tested if removal of one copy of *dpp* gene could suppress the germ cell differentiation defect. Interestingly, a copy of *dpp^hr4^* or *dpp^hr56^* can partially suppress the GSC-like tumorous phenotype caused by *egg* knockdown in ECs, and consequently more normal-looking germaria can be observed in comparison with *egg* knockdown alone (Figure 6A–6E). These results indicate that increased BMP signaling is at least in part responsible for the germ cell differentiation defect caused by *egg* knockdown in ECs. Recently, *Lsd1* has been suggested to repress *dpp* transcription in ECs, thus promoting germ cell differentiation [18]. One of the possibilities is that *egg* may be involved in repressing *dpp* transcription in ECs. To test the possibility, we used two independent RNAi strains against different regions of *dpp* to knock down *dpp* mRNA expression in the ECs in which *egg*RNAi is also expressed. *dpp* knockdown in ECs cannot rescue the germ cell differentiation defect caused by *egg* knockdown (Figure 6F–6H). In addition, our quantitative RT-PCR results also show that there is no increase in *dpp* mRNAs in the EC-specific *egg* knockdown ovaries (Figure S3A). These results indicate that *egg* is dispensable for *dpp* repression in ECs.

One recent study has clearly established that MAPK signaling functions downstream of EGFR in ECs to repress the expression of *dally*, whose gene product facilitates Dpp diffusion [42]. The *egg* mutant phenotype raises a possibility that *egg* is involved in the repression of *dally* expression in ECs. Interestingly, *dally* knockdown in ECs can partially suppress the germ cell differentiation defect caused by *egg* knockdown, indicating that *dally* upregulation in ECs contributes to the germ cell differentiation defect (Figure 6I–6K). To further test if *egg* is involved in regulation of EGFR signaling in ECs, we examined the expression of pERK, which has been used to monitor EGFR signal transduction in ECs [42], [45]. pERK is preferentially expressed in wild-type ECs as reported [45] (Figure 6L). In the *egg* knockdown ECs, pERK expression remains normal or close to normal (Figure 6M). These results suggest that *egg* functions either downstream of or in parallel with EGFR signaling to repress *dally* expression in escort cells.

### Egg Functions in ECs to Control Their Survival

**Figure 7:**
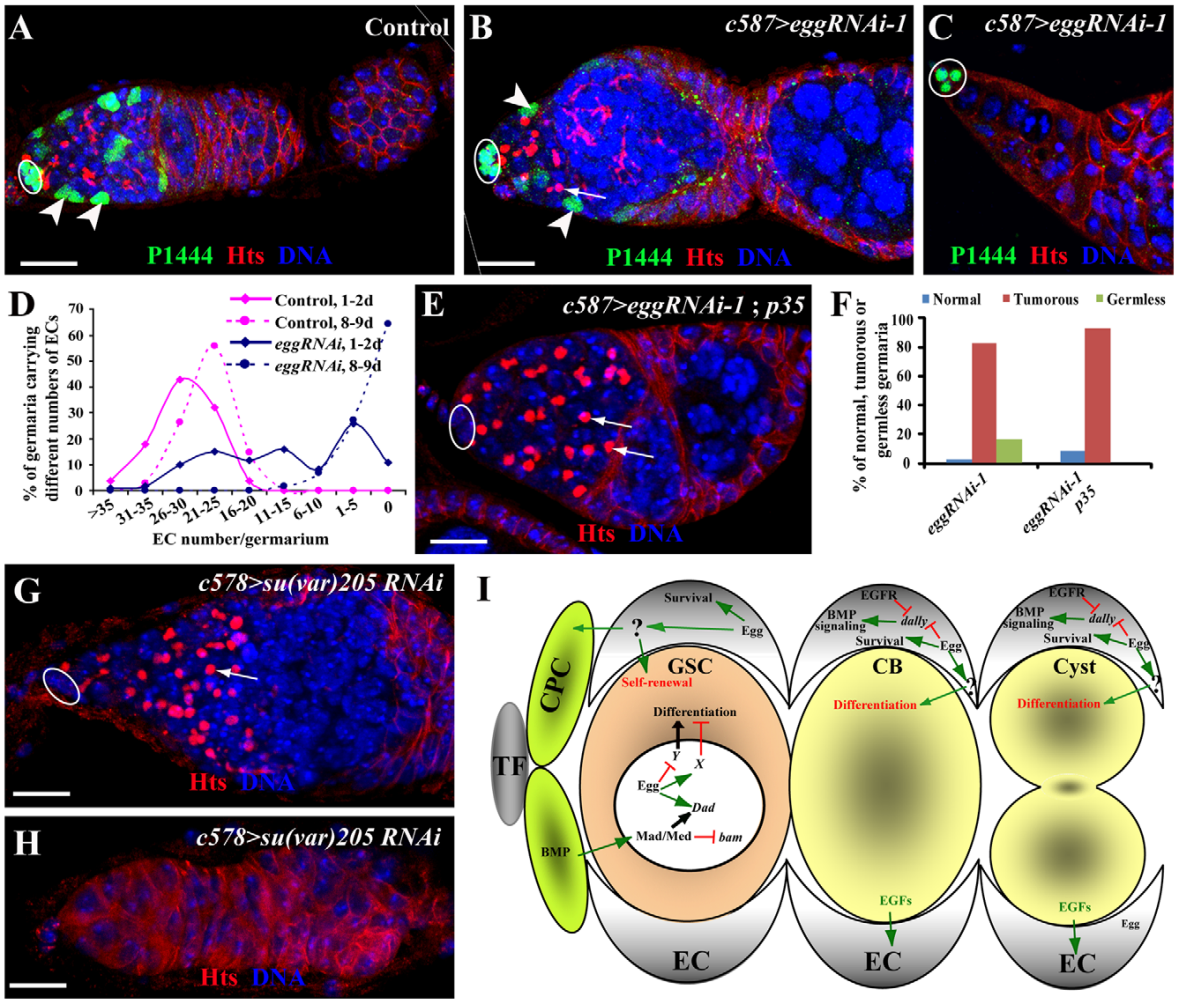
Egg is required for EC survival. (A) A control *PZ1444*/+ germarium shows *lacZ* expression in all the ECs (two by arrowheads) in addition to cap cells (oval). (B–D) *c587-gal4*-driven *egg* knockdown in ECs results in reduced ECs (two by arrowheads, B) or the complete absence of ECs (C), which are located posterior to cap cells (oval, B and C). D shows quantitative changes in EC numbers of control and *egg* knockdown germaria with age. (E, F) Overexpression of *p35* suppresses the EC loss caused by RNAi-mediated *egg* knockdown in ECs (arrows). Quantitative results (F) show that p35 overexpression in ECs suppresses the germless phenotype but not germ cell differentiation defects. (G, H) RNAi-mediated *su(var)205* knockdown in ECs causes the accumulation of spectrosome-containing single germ cells (arrow, G) and the complete loss of germ cells (H). (I) A working model for explaining functions of Egg in GSCs and ECs for controlling GSC maintenance and differentiation. In the GSC, Egg may repress the expression of a gene that is important for GSC differentiation or activate expression of a gene that is important for repressing GSC differentiation. In addition, it also directly or indirectly regulates *Dad* expression along with BMP signaling. In the EC, Egg is required for controlling the survival of the EC, which is important for proper germ cell differentiation and GSC maintenance. Egg may control expression of *dally* and other BMP regulators in the EC to prevent BMP signaling from spreading to differentiated germ cells.

During the characterization of the EC-specific *egg* knockdown mutant phenotype, we noticed that most of the *egg* knockdown germaria have smaller regions 1 and 2a than in the control germaria, while others appear to completely lose ECs, suggesting that *egg* knockdown in ECs leads to gradual EC loss (Figure 7A–7C). In the absence of ECs, germ cells are also depleted from the germaria (Figure 7C; Figure S4), suggesting that ECs are also required for maintaining GSCs. To further investigate if *egg* knockdown affects EC maintenance, we used the *lacZ* enhancer trap line *PZ1444* to quantify the number of ECs in wild-type and *egg* knockdown germaria. *PZ1444* is known to label both cap cells and ECs in the germarium [12], [46]. In the control germaria, *PZ1444* labels 20 to 35 ECs (Figure 7A and 7D). In the *egg* knockdown germaria, the number of ECs has already decreased at the age of 1–2 days (Figure 7D). At the age of 8 or 9 days, all *PZ1444*-positive ECs in 63% of the *egg* knockdown germaria are completely lost, and consequently no germ cells including GSCs exist in the germaria (Figure 7C and 7D). However, the egg chambers associated with those germaria are still full of spectrosome-containing single germ cells (Figure S4), indicating that germ cell differentiation is absolutely dependent on the presence of functional ECs. To further determine if EC loss is caused by apoptosis, we forced expression of *p35*, an apoptosis inhibitor, in the *egg* knockdown ECs. Indeed, *p35* expression can prevent EC loss and formation of germless germaria, indicating that Egg is required for maintaining EC survival and thus GSCs (Figure 7E and 7F). Interestingly, the germ cell differentiation defect remains, indicating that Egg also functions in ECs to promote germ cell differentiation through modulating BMP or other signaling pathways. These results suggest that *egg* is required for maintaining EC survival and regulating EC function for promoting germ cell differentiation.

### HP1 Is Required in ECs to Regulate Germ Cell Differentiation and Control EC Survival

To further determine if any other chromatin regulators are also required in ECs to regulate germ cell differentiation, we sought to use the same RNAi approach to knock down expression of *sin3A*, *su(z)12* and *su(var)205* genes, which encode general factors regulating heterochromatin formation or repressing gene transcription [29], [47], [48]. Interestingly, knockdown of *sin3A* and *su(z)12* in ECs fails to yield any discernible phenotype on germ cell differentiation. In contrast, knockdown of *su(var)205* in ECs leads to the germ cell differentiation defect and the EC loss phenotype, which is identical to those in *egg* knockdown (Figure 7G and 7H). This is consistent with the biochemical function of Su(var)205, a HP1 protein binding to H3K9me3 [19], [49], [50], [51]. These results suggest that HP1 and Egg, but not other general transcriptional repressors, function specifically in ECs to control EC survival and regulate germ cell differentiation.

## Discussion

Although the mouse H3K9 trimethylase SETDB1 was recently shown to be important for maintaining ESC self-renewal by repressing the expression of developmentally regulated genes [8], its role in regulation of adult stem cells has not yet been established. In this study, we show that the *Drosophila* SETDB1 homolog, Egg, is required intrinsically for controlling GSC self-renewal and extrinsically for controlling GSC differentiation in the *Drosophila* ovary. The *egg* mutant ovaries exhibit both GSC loss and germ cell differentiation defects. We further demonstrate that Egg controls GSC self-renewal by repressing a Bam/Bgcn-independent pathway. In addition, EC-specific RNAi-mediated knockdown of *egg* function leads to gradual EC loss and germ cell differentiation defects, indicating that Egg is required for EC maintenance and germ cell differentiation. Recently, we have proposed that ECs function as a niche for promoting germ cell differentiation [52]. Furthermore, Egg functions in ECs to control germ cell differentiation at least in part by preventing BMP signaling from spreading to the differentiation niche and regulating EC survival. Therefore, we propose that Egg is a key H3K9 trimethylase in the *Drosophila* ovary, which is required intrinsically for controlling GSC self-renewal via repressing a Bam/Bgcn-independent differentiation pathway and in ECs for controlling germ cell differentiation by preventing BMP signaling spreading to the differentiation niche (Figure 7I). The findings from this study have further supported the idea that ECs function as a germ cell differentiation niche. It will be of great interest to test if SETDB1 is also important for controlling adult stem cell self-renewal and differentiation in mammalian systems.

### Egg Is Required Intrinsically for Controlling GSC Self-Renewal by Repressing a Bam/Bgcn-Independent Differentiation Pathway

In the previous study [28], Egg was shown to be a primary H3K9 trimethylase in follicle progenitor cells for maintaining H3K9me3 and regulating their proliferation and survival [28]. Egg and its co-factor Wde were also shown to be required for maintaining H3K9me3 in early germ cells and regulating their survival [29]. This study has further demonstrated that Egg is required intrinsically for controlling GSC self-renewal and proliferation. Consistent with the previous finding [28], we have shown that H3K9me3 but not H3K9me2 is eliminated in marked *egg* mutant GSCs. In addition, marked *egg* mutant GSCs are lost rapidly from the niche in comparison with the marked control GSCs, further supporting the idea that Egg is required for GSC maintenance. Moreover, the marked *egg* mutant GSCs and mitotic cysts are negative for TUNEL-based ApopTag labeling, but the marked 16-cell cysts in the regions 2b and 3 of the germarium are observed to be positive, indicating that Egg is dispensable for the survival of GSCs and early mitotic cysts but is required for the survival of 16-cell cysts. Finally, marked *egg* mutant GSCs appear to proliferate slower than the control GSCs based on cyst production and BrdU labeling. We used RNAi-mediated knockdown to show that loss of Egg function from GSCs and their progeny leads to the accumulation of DNA damage, suggesting that Egg is required for maintaining genome integrity. The accumulated DNA damage could also explain retarded GSC proliferation and increased 16-cell cyst apoptosis. These results demonstrate that Egg is required intrinsically for GSC self-renewal and proliferation and for the survival of 16-cell cysts.

BMP signaling and E-cadherin-mediated cell adhesion are essential for maintaining GSCs in the *Drosophila* ovary [11], [13], [14], [33]. BMP signaling represses *bam-GFP* expression and activates *Dad-lacZ* expression in GSCs [13], [14], [35], [36]. H3K9me3 is thought to be a histone marker for heterochromatin formation and transcriptional repression [1]. Surprisingly, in marked *egg* mutant GSCs, *bam-GFP* remains repressed as in wild-type GSCs, but *Dad-lacZ* expression fails to be activated, indicating that Egg, and presumably H3K9me3, is dispensable for BMP signaling-mediated transcriptional repression of *bam*. The requirement of Egg for transcriptional activation of *Dad* could be indirect, but the detailed mechanism awaits further investigation. We have further demonstrated functionally that Egg controls GSC self-renewal by repressing a Bam/Bgcn-independent pathway by showing that marked *bgcn egg* double mutant GSCs are still lost at a much faster rate than marked control GSCs. Previously, Pumilio and Pelota were proposed to control GSC self-renewal by repressing a Bam/Bgcn-independent differentiation pathway as mutations for either factor can drive differentiation of *bam* mutant germ cells [53]–[55]. Interestingly, mutations in *egg* can also cause differentiation of *bgcn* mutant germ cells, further supporting the idea that Egg represses a Bam/Bgcn-independent differentiation pathway to maintain GSC self-renewal. There are two possible strategies for Egg to repress differentiation and thus maintain GSC self-renewal: Egg represses the expression of a gene(s) important for GSC differentiation or activates the expression of a gene(s) critical for repressing GSC differentiation (Figure 7I). Unfortunately, it remains unclear how Egg represses GSC differentiation to maintain self-renewal. Therefore, the identification of Egg target genes in GSCs will help define the unknown GSC differentiation pathway along with the identification of target genes of Pumilio and Pelota in order to gain a deeper understanding of GSC self-renewing mechanisms.

During the revision of this manuscript, a study was published to propose that Egg is required for H3K9me3 and heterochromatin formation in CBs and differentiated cysts, and is required for expression of piRNA genes and thus repression of transposable elements (TEs) [56]. Loss of piRNAs in germ cells is known to cause the activation of transposable elements (TEs) and consequently an increase in DNA damage [57]. Consistently, our study shows that loss of *egg* function in germ cells leads to the accumulation of DNA damage. The regulation of piRNA by Egg offers mechanistic insight into why Egg is required for GSC maintenance and proliferation [56]. However, our study has two different findings. One is that H3K9me3 establishment begins from GSCs, but not from CBs as the published study proposed [56]. The other is that Egg is also required intrinsically for GSC maintenance and proliferation, but not for CB differentiation. The published study showed that spectrosome-containing single germ cells accumulate following germline-specific *egg* knockdown [56]. In our study, germline-specific expression of *eggRNAi-1* leads to GSC loss, which is consistent with our mutant clonal analysis results, whereas the expression of *eggRNAi-2* results in swollen germaria containing a few more spectrosome-containing CBs and cysts than control. The accumulation of the few more single germ cells is likely due to DNA damage-caused slowdown of mitotic progression. The difference between the published study and our study could be simply caused by different *egg* knockdown efficiencies.

### Egg Controls Germ Cell Differentiation by Regulating EC Survival and BMP Signaling

*egg* homozygous ovaries accumulate more undifferentiated germ cells and gradually lose their GSCs, which appear to be paradoxical. The *egg* mutant GSC loss phenotype can be attributed to the intrinsic requirement for GSC self-renewal. Our further genetic analysis has revealed the requirement of Egg in ECs for controlling GSC differentiation by EC-specific RNAi-mediated *egg* knockdown. In the absence of Egg function from ECs, GSC progeny fail to differentiate and continuously proliferate as single germ cells, indicative of differentiation defects. In addition, loss of Egg function in ECs also causes EC loss, and in the complete absence of ECs, the progeny that have been generated before GSC loss also accumulate as single germ cells, further supporting that ECs are required for CB differentiation. Some of the accumulated single germ cells appear to upregulate *Dad-lacZ* expression and repress *bam-GFP* expression, suggesting that BMP signaling spreads to the germ cell differentiation niche, thereby interfering with germ cell differentiation. These findings suggest that Egg is required in ECs to promote germ cell differentiation at least in part by preventing self-renewal-promoting BMP signaling from spreading to the germ cell differentiation niche.

EFGR signaling has been suggested to act in ECs to control germ cell differentiation by repressing expression of Dally, a protein important for facilitating BMP diffusion [42]. Interestingly, in the *egg* knockdown ECs, the expression of pERK, an EGFR signaling indicator, still remains normal, indicating that Egg is not essential for EGFR signaling in ECs. However, *dally* knockdown in ECs can partially suppress the *egg* knockdown mutant germ cell tumor phenotype, indicating that upregulation of *dally* in *egg* knockdown ECs contributes to BMP upregulation in the differentiation niche and to germ cell differentiation defects. The regulation of *dally* in ECs by Egg could be direct or indirect. The newly published study on Egg has shown that loss of Egg function in ECs leads to defective piRNA production and germ cell differentiation defects [56]. Consistently, we also confirmed that *egg* knockdown in ECs results in dramatically increased expression of TEs (Figure S3B and S3C). The germ cell differentiation defect can be rescued by a mutation in one of the DNA damage checkpoint genes, suggesting that DNA damage in ECs affects their ability to regulate germ cell differentiation [56]. It will be of great interest to investigate if the mutation in the checkpoint gene also rescues defective BMP signaling in differentiated cells. Based on our findings from this study, we propose that Egg functions downstream of or in parallel with EGFR signaling to repress *dally* expression in ECs, thereby preventing BMP signaling from spreading to the differentiation niche (Figure 7I). Because the signal(s) from ECs to control germ cell differentiation has not been identified yet, it remains unclear whether Egg also regulates additional factors independent of BMP signaling in ECs to control germ cell differentiation.

### GSC-Contacting ECs Function as an Integral Part of the GSC Niche

In this study, we have also shown that the *egg* knockdown ECs are gradually lost, and that GSCs cannot be maintained in the complete absence of ECs. This is consistent with our recently published finding that disruption of Rho function in ECs also cause EC loss and thus GSC loss [58]. Because 5 to 6 most anteriorly localized ECs directly contact cap cells and GSCs, we propose that these ECs function as a part of the GSC niche to promote self-renewal by directly providing signals or indirectly by regulating cap cells function (Figure 7I). One previous study suggests that JAK-STAT signaling functions in ECs to control GSC maintenance indirectly [59]. How these GSC-contacting ECs contribute to GSC regulation remains to be further investigated.

## Materials and Methods

### *Drosophila* Stocks

Flies were maintained at 25°C on molasses-based media supplied with live yeast unless otherwise specified. The strains used in this study include: *egg^235^*, *egg^2138^* and *egg^1473^* [28](kindly provided by T. Hazelrigg); *Dad-lacZ* [60], *bam-GFP* [61], *PZ1444* [46], *nos-gal4* [62], *c587-gal4* [63], *dpp^hr56^* [11], *dpp^hr4^* [11], *UAS-p35* [64] and *w^1118^* (control).

### RNAi-Mediated *egg* Knockdown in ECs

For *egg* knockdown in ECs, the *c587-gal4*; *UAS-dcr2* line was used to drive the expression of five independent *eggless* RNAi constructs, HMS00443 and HMS00112 from Harvard Medical School (kindly provided by Dr. N. Perrimon), and three other lines (#21172, #33730 and #101677) from the Vienna *Drosophila* RNAi Center (VDRC). *UAS-RNAi* lines for *dpp* (*dppRNAi-1*: JF01090; *dppRNAi-2*: JF01371)and *dally* [52] were kindly provided by Dr. N. Perrimon and Dr. X. Lin (Cincinnati Children's Hospital Medical Center), respectively. After eclosion, their progeny were collected and reared at 29°C for several days as described in the text for each experiment.

### Generation of Marked *egg* Mutant GSC Clones

The marked control and *egg* mutant GSC clones were generated using the FLP-mediated FRT recombination technique as described previously [11], [33]. H3K9me3, H3K9me2 and TUNEL staining were performed 7 or 12 days after clone induction (ACI). The genotypes used for clonal analysis were: (1) *hs-flp/+*; *FRT_42D_/FRT_42D_ ubiGFP*; (2) *hs-flp/+*; *FRT_42D_ egg^235^/FRT_42D_ ubiGFP*; (3) *hs-flp/+*; *FRT_42D_ egg^1473^/FRT_42D_ ubiGFP*, (4) *hs-flp/+*; *FRT_42D_ egg^2138^/FRT_42D_ ubiGFP*, *(5) hs-flp/+;FRT_42D_/FRT_42D_ arm-lacZ*; *(6) hs-flp/+*; *FRT_42D_ egg^235^/FRT_42D_ arm-lacZ;(7) hs-flp/+*; *FRT_42D_ egg^1473^/FRT_42D_ arm-lacZ and (8) hs-flp/+*; *FRT_42D_ egg^2138^/FRT_42D_ arm-lacZ*. For generating *bgcn egg* double mutant clones, the following genotypes were used: *(1) hs-flp/+*; *FRT_42D_ bgcn^20915^ egg^235^/FRT_42D_ arm-lacZ*; *(2) hs-flp/+*; *FRT_42D_ bgcn^20915^ egg^1473^/FRT_42D_ arm-lacZ*.

### Immunohistochemistry and Fluorescent Microscopy

Ovaries were dissected, fixed and stained according to the method described previously [33]. The following primary antibodies were used: monoclonal mouse anti-Hts (1B1, 1∶4, DSHB), mouse anti-Lamin C (LC28.26, 1∶4, DSHB), mouse anti-Orb (4H8, 1∶4, DSHB), mouse anti-Fas3 (7G10, 1∶3, DSHB), rat anti-Vasa (1∶10, DSHB), rat anti-E-cadherin DCAD2 (1∶3, DSHB), polyclonal rabbit anti-GFP (1∶100, Molecular Probes), chicken anti-GFP antibody (1∶200, Invitrogen), rabbit anti-β-galactosidase (1∶100, Molecular Probes), rabbit anti-H3K9me3 (1∶200, Abcam ab8898) and rabbit anti-phosphorylated ERK1/2 (1∶200, a gift from Dr. Y. Cai). Secondary antibodies used were: goat anti-rabbit, goat anti-rat and goat anti-mouse antibodies conjugated to Alexa 488, Alexa 568 or Cy5 (1∶100, Molecular Probes) and Dylight 488 donkey anti-chicken antibody (1∶200, Jackson ImmunoResearch Laboratories). TUNEL staining was performed using the ApopTag Red *In Situ* Apoptosis Detection Kit (Chemicon, S7165) according to the manufacturer's protocol. All micrographs were taken using an inverted Leica TCS SP5 confocal microscope. For the experiments involving comparison between mutants and wild type, the same parameters were used for confocal imaging.

## Supporting Information

Figure S1 Germline-specific *egg* knockdown leads to GSC loss and DNA damage accumulation. (A) A week-old control germarium contains three GSCs (arrows) and differentiated germ cells. (B) *nos-gal4*-driven expression of *eggRNAi-1* leads to complete depletion of germ cells including GSCs in the majority of the week-old germaria. (C) Quantitative results of germless germaria following expression of *eggRNAi* in week-old females. (D) A week-old control germarium contains branched fusome-containing differentiated germ cells (arrow) and H2AX-positive meiotic germ cells. (E) *nos-gal4*-driven expression of *eggRNAi-2* leads to the accumulation of extra spectrosome-containing single germ cells (arrowheads) and branched fusome-containing differentiated germ cells, which are positive for H2AX.
(TIF)

Figure S2 *egg* knockdown in ECs leads to accumulation of spectrosome-containing single germ cells. Four independent *egg* RNAi lines, which are targeted to different regions of the *egg* transcript, generate similar germ cell differentiation defects following their expression in ECs using the *c587 gal4* driver.
(TIF)

Figure S3 EC-specific *egg* knockdown leads to upregulation of transposable elements *gypsy* and *tart* but not *dpp*. These quantitative RT-PCRs are normalized to multiple internal gene controls including *actin42A*, *rp49* and *gapdh*, while the value for the *c587* driver control is designated to 1. All these results are based on two independent experiments. (A) *dpp* mRNA expression shows little change following *c587*-driven expression of either *eggRNAi-1* or *eggRNAi-2*. (B, C) *gyspy* (B) and *tart* (C) mRNA expression shows dramatic changes following *c587*-driven expression of either *eggRNAi-1* or *eggRNAi-2*.
(TIF)

Figure S4 ECs are required for GSC maintenance and germ cell differentiation. In a germarium in which *egg* function is knocked down through RNAi, GSCs are already lost from the niche (circle), while most of the progeny of the lost GSCs remain as single germ cells indicated by spectrosomes (arrows).
(TIF)
