# Supplementary figures and images for: Histone H3K9 Trimethylase Eggless Controls Germline Stem Cell Maintenance and Differentiation
Source: PLoS Genet. 2011 Dec 22;7(12):e1002426. doi: 10.1371/journal.pgen.1002426 (PMC3245301; doi:10.1371/journal.pgen.1002426)

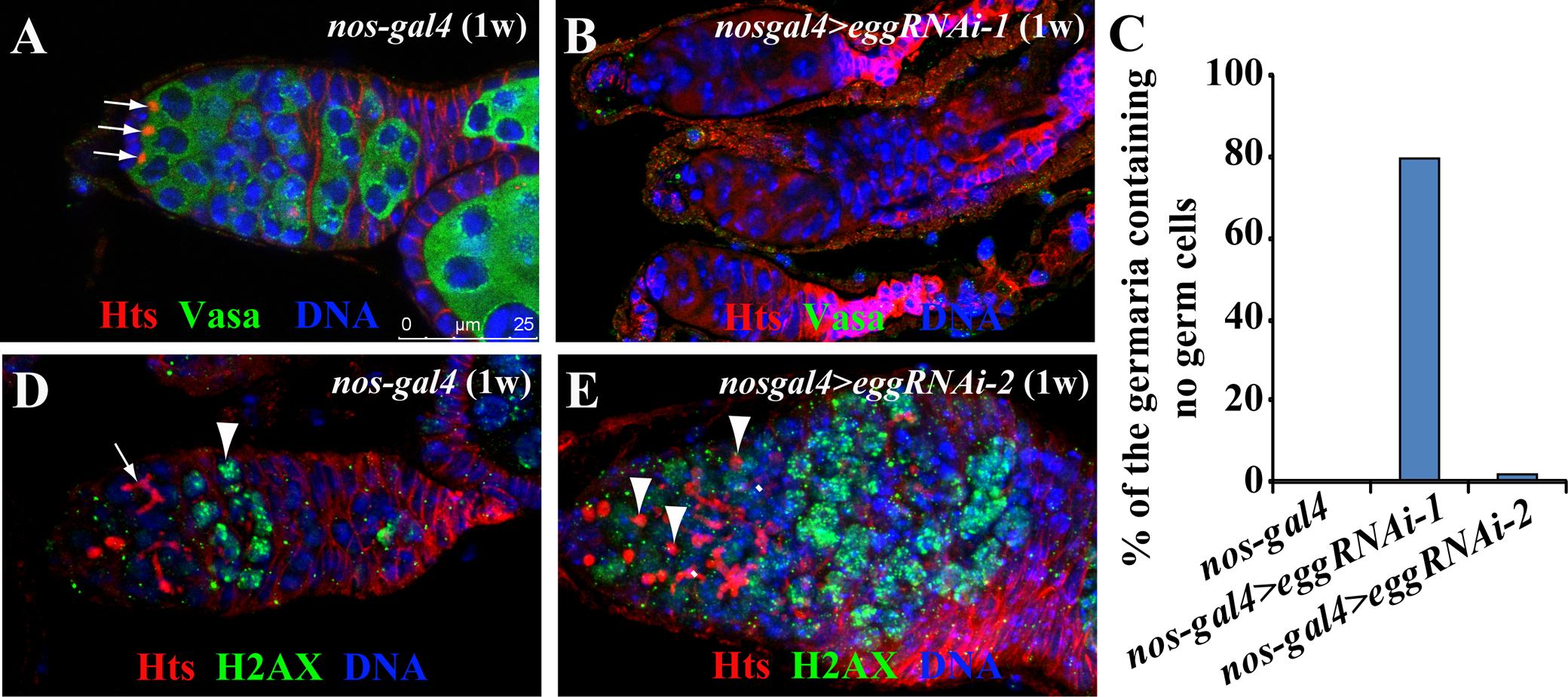

Supplement: Figure S1 — Germline-specific egg knockdown leads to GSC loss and DNA damage accumulation. (A) A week-old control germarium contains three GSCs (arrows) and differentiated germ cells. (B) nos-gal4-driven expression of eggRNAi-1 leads to complete depletion of germ cells including GSCs in the majority of the week-old germaria. (C) Quantitative results of germless germaria following expression of eggRNAi in week-old females. (D) A week-old control germarium contains branched fusome-containing differentiated germ cells (arrow) and H2AX-positive meiotic germ cells. (E) nos-gal4-driven expression of eggRNAi-2 leads to the accumulation of extra spectrosome-containing single germ cells (arrowheads) and branched fusome-containing differentiated germ cells, which are positive for H2AX. (TIF) [file pgen.1002426.s001.tif]

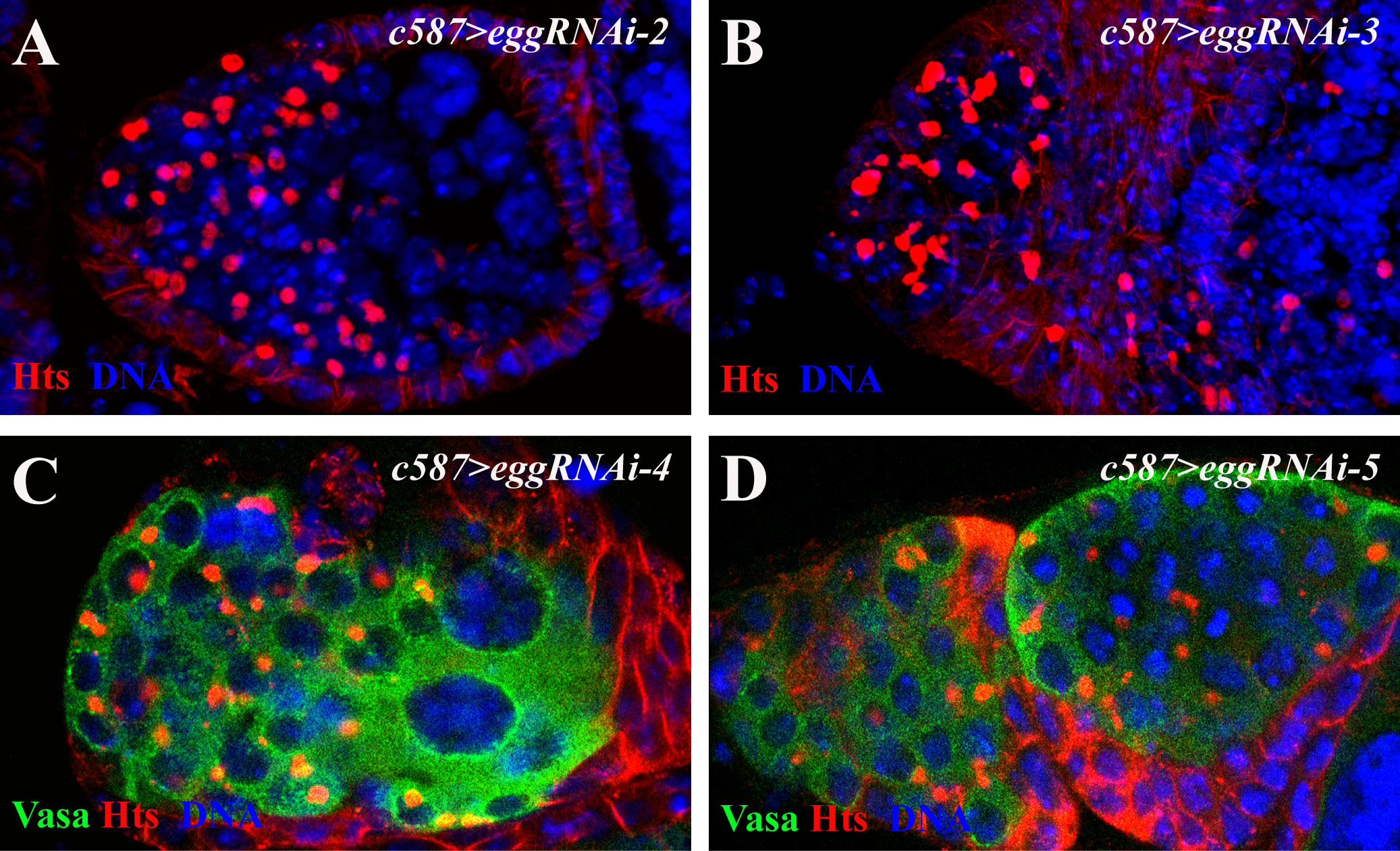

Supplement: Figure S2 — egg knockdown in ECs leads to accumulation of spectrosome-containing single germ cells. Four independent egg RNAi lines, which are targeted to different regions of the egg transcript, generate similar germ cell differentiation defects following their expression in ECs using the c587 gal4 driver. (TIF) [file pgen.1002426.s002.tif]

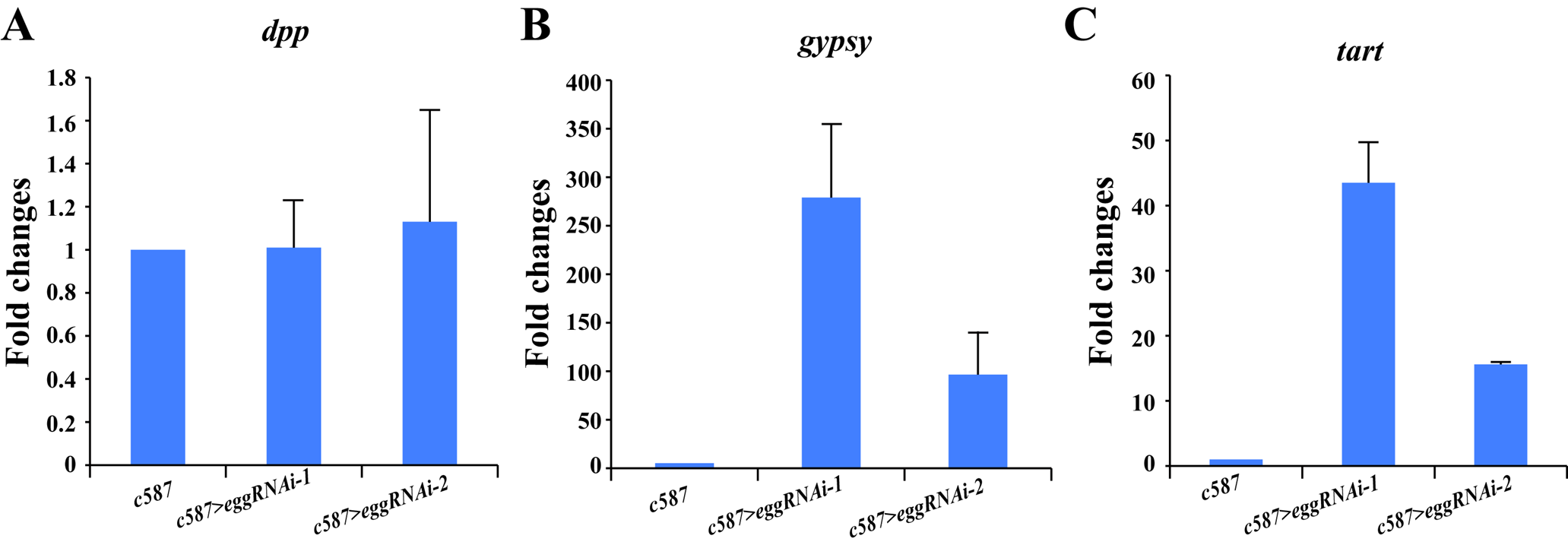

Supplement: Figure S3 — EC-specific egg knockdown leads to upregulation of transposable elements gypsy and tart but not dpp. These quantitative RT-PCRs are normalized to multiple internal gene controls including actin42A, rp49 and gapdh, while the value for the c587 driver control is designated to 1. All these results are based on two independent experiments. (A) dpp mRNA expression shows little change following c587-driven expression of either eggRNAi-1 or eggRNAi-2. (B, C) gyspy (B) and tart (C) mRNA expression shows dramatic changes following c587-driven expression of either eggRNAi-1 or eggRNAi-2. (TIF) [file pgen.1002426.s003.tif]

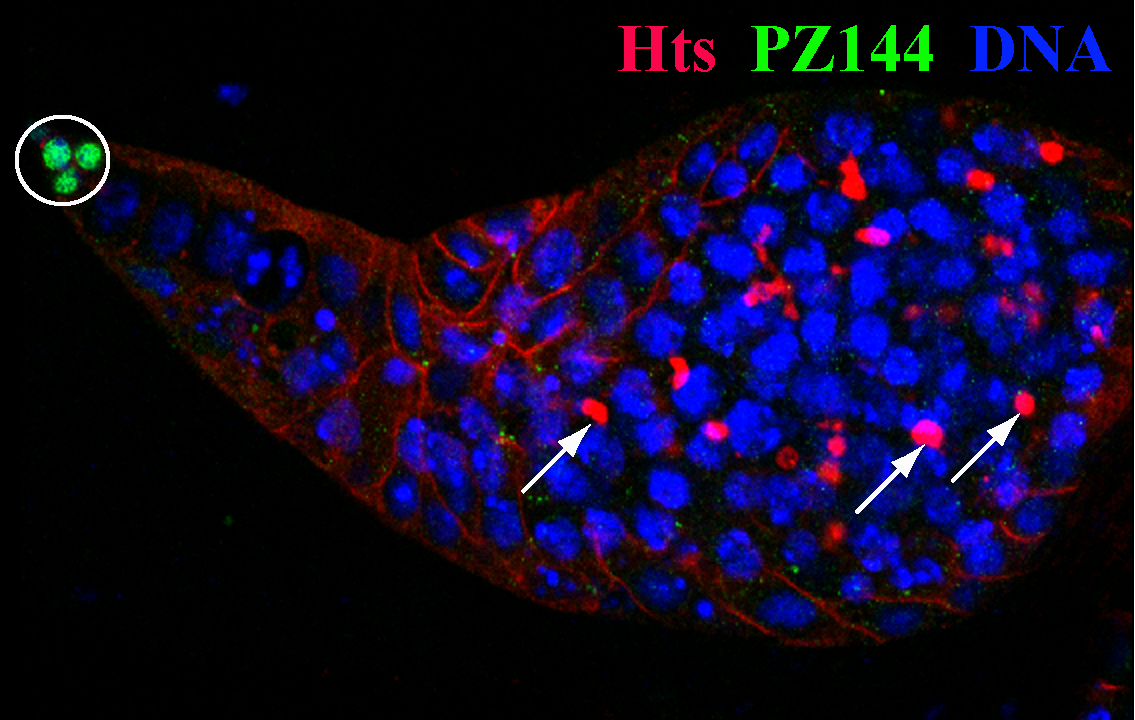

Supplement: Figure S4 — ECs are required for GSC maintenance and germ cell differentiation. In a germarium in which egg function is knocked down through RNAi, GSCs are already lost from the niche (circle), while most of the progeny of the lost GSCs remain as single germ cells indicated by spectrosomes (arrows). (TIF) [file pgen.1002426.s004.tif]
